# Comparison of Antimicrobial Properties of Graphene Oxide-Based Materials, Carbon Dots, and Their Combinations Deposited on Cotton Fabrics

**DOI:** 10.3390/ijms25105328

**Published:** 2024-05-14

**Authors:** Zakhar Ivanovich Evseev, Lidia Andreevna Tarasova, Fedora Dmitrievna Vasilieva, Marfa Nikitichna Egorova, Petr Stanislavovich Dmitriev, Yana Aleksandrovna Akhremenko, Svetlana Afanasyevna Smagulova

**Affiliations:** 1 Institute of Physics and Technologies, North-Eastern Federal University, 677000 Yakutsk, Russia; zi.evseev@s-vfu.ru (Z.I.E.); mn.egorova@s-vfu.ru (M.N.E.); sa.smagulova@s-vfu.ru (S.A.S.); 2 Medical Institute, North-Eastern Federal University, 677000 Yakutsk, Russiayaa.akhremenko@s-vfu.ru (Y.A.A.)

**Keywords:** graphene oxide, electrochemically exfoliated multigraphene, carbon dots, antimicrobial textiles, antimicrobial nanomaterials

## Abstract

The rise in the antibiotic resistance of bacteria has increased scientific interest in the study of materials with unique mechanisms of antimicrobial action. This paper presents the results of studies on the antimicrobial activity of carbon materials and textiles decorated with them. A comparative analysis of the bactericidal and fungicidal activities of graphene oxide, electrochemically exfoliated multigraphene, carbon dots, and their combinations was performed. Microbiological studies on reference strains of *E. coli*, *S. aureus*, and *C. albicans* showed that graphene oxide inhibited growth with up to 98% efficiency. Electrochemically exfoliated multigraphene was less effective (up to 40%). This study found no significant antimicrobial activity of carbon dots and the combination of carbon dots with graphene oxide significantly weakened their effectiveness. However, the combination of electrochemically exfoliated multigraphene and carbon dots exhibits a synergistic effect (up to 76%). A study on the antimicrobial activity of decorated cotton textiles demonstrated the effectiveness of antimicrobial textiles with graphene oxide, electrochemically exfoliated multigraphene, and a combination of carbon dots with electrochemically exfoliated multigraphene.

## 1. Introduction

Antibiotics are widely used in medicine, agriculture, and other fields of human activity. Many bacteria develop resistance to antibiotics over time via mutations. To solve this problem, a search is being carried out for new antimicrobial materials with different mechanisms of action, to which the development of resistance in bacteria is less likely. Currently, carbon nanomaterials such as carbon nanotubes [1], graphene oxide (GO) [2], and carbon dots (CDs) [3], which have unique antimicrobial activity, have attracted much attention.

Graphene is a monolayer of sp^2^ hybridized carbon atoms packed into a hexagonal structure [4]. GO is graphene containing various oxygen functional groups attached to its surface [5]. Nanoparticles exhibit different mechanisms of bacterial inhibition, such as cell rupture, protein denaturation, organelle dysfunction, DNA fragmentation, and inhibition of the electron transfer chain [6]. These effects result in disruption of cellular integrity and leakage of cellular content. The mechanisms of interaction of graphene and GO with bacteria are diverse and include physical and chemical effects. There are two types of physical effects: cutting bacteria with sharp edges of graphene [7] and wrapping bacterial cells with GO flakes [8]. Chemical effects include oxidative stress [9], which is caused by the release of reactive oxygen species during reactions between the functional groups of GO and bacterial membrane molecules and organelles. Investigations on the antimicrobial activity of GO have shown conflicting results owing to the influence of many factors [10]. Studies have shown the antibacterial activity of GO against both gram-positive (*S. aureus* [11] and *E. faecalis* [12]) and gram-negative bacteria (*E. coli* [13] and *P. aeruginosa* [14]). It was shown in [15] that the larger the lateral dimensions of GO, the more pronounced the antimicrobial effect is. With an increase in the area of GO flakes from 0.010 μm^2^ to 0.753 μm^2^, the viability of *E. coli* decreased from 46% to 98%. It was concluded that the main mechanism for the suppression of gram-negative *E. coli* growth was the wrapping of bacteria in GO flakes, which led to membrane rupture. Another study claimed [12] that GO has higher adhesion to gram-positive bacteria (*S. aureus* and *E. faecalis*) and the mechanism of cell wrapping is more pronounced in this case. For gram-negative bacteria (*E. coli* and *P. aeruginosa*), the main mechanism is mechanical damage by the sharp edges of GO flakes [12]. In a study [16], it was shown that the surface morphology of wrinkled GO had an effect on its antimicrobial properties. When the curvature of GO coincides with the size of the bacteria, the antibacterial effect is more pronounced. In another study [11], the effect of GO flake aggregation on antimicrobial activity was evaluated. When inorganic salts are added to aqueous suspensions with high concentrations of GO, macrosized agglomerates are formed, which, in contrast, promote the growth of bacterial colonies. In addition, the composition and concentration of the oxygen groups located on the surface of the GO flakes play an important role. Some studies have shown that GO has higher antimicrobial activity [17,18] than reduced GO, which has a lower oxygen content, and in others, vice versa [19,20]. This discrepancy may be due to differences in research methods as well as in the concentration and quality of the initial GO. For example, in [21], it was shown that commercially available GO did not exhibit antibacterial properties after thorough washing with deionized water. The authors concluded that the antimicrobial properties were mainly due to the acidic and other contaminants remaining after GO synthesis. However, the disappearance of the antimicrobial properties of GO washed with deionized water may be due to aggregation of the GO flakes.

Another field of interest is the antimicrobial properties of CDs and the synergistic effect of GO and CD composites. CDs are zero-dimensional carbon nanoparticles with dimensions of ~10 nm, consisting of crystalline cores and various functional groups on their surfaces [22]. CDs showed high antimicrobial activity and, at the same time, had low cytotoxicity relative to mammalian cells [3]. Functional groups play a major role in the antimicrobial activity of CDs [23]. For example, in [24], it was shown that functionalization of CDs with nitrogen groups increased the antimicrobial effect against gram-positive *B. subtilis* compared to sulfur-doped CDs. However, for gram-negative *E. coli*, the antimicrobial effects of CDs doped with nitrogen and sulfur are the same. The authors attributed this effect to the influence of the type of charge on the surface of the CDs and the negatively charged shell of bacteria.

The development of effective, environment-friendly, and wash-resistant antimicrobial textiles is an important problem. The deposition of nanoparticles on the surfaces of fabrics leads to various challenges, such as uniformity of the deposited nanoparticle films, washing resistance, and disruption of some of the mechanisms of inhibition, such as cell wrapping [25,26,27,28]. The development of graphene-based antimicrobial textiles that effectively suppress the growth of bacteria is in demand in medicine and hygiene. A study [29] showed that cotton textiles decorated with GO exhibited pronounced antimicrobial properties. It can inactivate up to 98% of bacteria before washing and up to 90% of bacteria after multiple wash cycles. The antimicrobial activity of GO-coated textiles and GO suspensions showed better toxicity toward gram-positive bacteria than gram-negative bacteria [30].

The conflicting results on the antimicrobial properties of GO require further studies aimed at identifying the relationship between the morphology, structure, and chemical composition of GO and its antimicrobial properties. Of particular interest is the antimicrobial activity of mildly oxidized multigraphene (MOG), synthesized by the electrochemical exfoliation of graphite [31]. This method allows the production of large quantities of material and is more suitable for mass production. In [32], the antimicrobial properties of MOG obtained from highly oriented pyrolytic graphite were studied. MOG did not show a significant antimicrobial effect. To our knowledge, this is the only study in which the antimicrobial properties of electrochemically exfoliated MOG were investigated. In addition, the antimicrobial activity of the GO/CDs combination has not yet been studied. This field is of particular interest because the cross-linking of Ag nanoparticles with GO leads to a synergetic antimicrobial effect [33]. Investigation of the antimicrobial properties of GO, MOG, CDs, and their combination on the surface of cotton textiles could help to evaluate their effectiveness as antibacterial impregnating agents.

This study compares the antimicrobial activity of GO synthesized by two different methods: The Hummers method, which produces GO with a high oxygen content, and the electrochemical exfoliation method, which synthesizes MOG with a low oxygen content. Comparison of GO and MOG can reveal the influence of oxygen content and morphology of the oxidized graphene/multigraphene flakes on antimicrobial activity. For the first time, the antifungal activity of MOG was evaluated, as well as the antimicrobial activity of GO/CDs and MOG/CDs combinations. The antimicrobial effectiveness of cotton textiles decorated with GO, MOG, and CDs and their combinations were studied. The obtained data can be used for the development of effective antimicrobial textiles for medicine, smart clothing, and other applications.

## 2. Results

### 2.1. Characterisation of Materials

**Figure 1:**
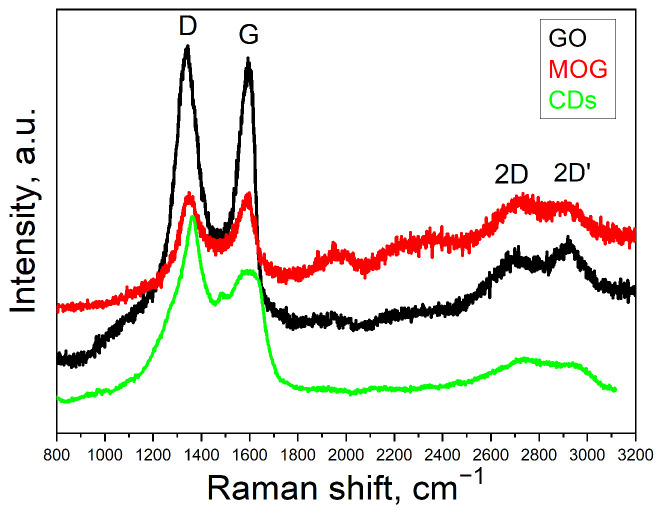
Raman spectra of GO, MOG, and CDs.

The Raman spectra of GO, MOG, and CDs are shown in Figure 1. The spectra contain four peaks: D, G, 2D, and 2D’. The D- and G-peaks localized at ~1350 cm^−1^ and ~1580 cm^−1^, respectively, are typical of graphite/graphene materials [34]. The G-peak is associated with the first-order Raman mode, E_2g_. Peak D is associated with the A_1g_ breathing mode, which represents defects in the crystal lattice plane, edge plane density, and sp^3^-hybridized carbon atoms [35]. In our case, shifts to the regions of low (1345 cm^−1^) and high frequencies (1613 cm^−1^) of the D and G peaks, respectively, indicate a high degree of lattice disorder, which correlates with the literature data for GO [36]. The D and G peaks of the carbon materials make it possible to indirectly estimate the ratio of sp^3^/sp^2^-hybridized carbon atoms. In the case of GO, the higher intensity of the D-peak compared to that of MOG can be associated with a higher oxygen content [36]. The density of defects can be estimated from the ratio of the integral intensities of the I(D) and I(G) bands [34]. I(D)/I(G) was determined according to [37], which amounted to 1.6 for GO and MOG, indicating the same density of defects in both materials. In the case of the CDs, the G peak is shifted to lower frequencies (1550 cm^−1^) and its peak is very broad compared to those of GO and MOG. The I(D)/I(G) ratio of the CDs is very high, indicating a high degree of disorder in the crystal lattice [36]. In addition, there is a two-phonon band localized near the ~2700 cm^−1^, called the 2D peak [38]. This band represents second-order Raman scattering arising from the breathing mode of carbon rings in the crystal lattice plane [37]. This peak provides information on the crystallinity of the graphite or graphene layers [38]. The low intensity of the 2D peak of all carbon nanomaterials can be attributed to the functional groups, which leads to a low density of sp^2^ carbon crystallites. Peak 2D’ localized in the ~2900 cm^−1^ superimposed with peak 2D indicates the number of defects in the carbon structure and the presence of amorphous carbon [39]. The weak intensities of the 2D and 2D’ peaks in the Raman spectrum of the CDs compared to those of GO and MOG indicate a lower density of sp^2^ crystallites.

The results of the elemental composition analysis by energy dispersive spectroscopy (EDS) of the synthesized materials are presented in Table 1. The highest oxygen content (41.4%) was observed for GO. The atomic oxygen content of the MOG was 6.1%. The oxygen content of the CDs was 33.5%. In addition, a fairly large number of nitrogen atoms were found (28.8%).

**Figure 2:**
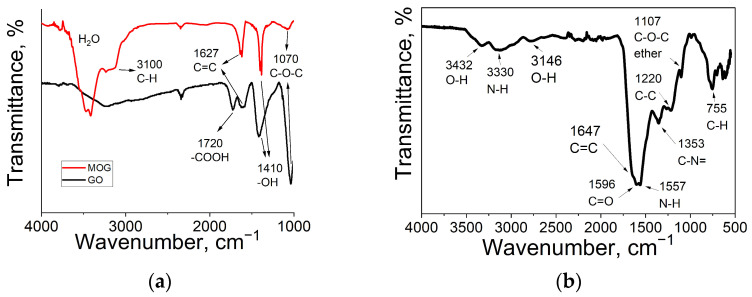
IR spectra of (**a**) GO and MOG and (**b**) CDs.

Peaks corresponding to various oxygen functional groups were detected in the infrared (IR) spectra of the MOG and GO (Figure 2a). The spectra show absorption maxima in the region of ~3200–3400 cm^−1^, corresponding to the vibrations of hydroxyl groups. Peaks corresponding to the stretching vibrations of O-H bonds of hydroxyl groups, 1410 cm^−1^, vibrations of epoxy and other C-O groups, 1070 cm^−1^, and a peak corresponding to carbon bonds in sp^2^ hybridization at 1627 cm^−1^ are present [40]. The main difference between MOG and GO is the presence of vibration peaks of the C=O bonds of carboxyl groups (-COOH) at the edges of the layer planes in the region of 1720 cm^−1^. In addition, peaks associated with the stretching vibrations of alkene groups C-H in the region of 3100 cm^−1^ are present in MOG. In the IR spectrum of the CDs (Figure 2b), the peaks at 1645 cm^−1^, 1596 cm^−1^, and 1350 cm^−1^ are attributed to the stretching vibrations of C=N/C=O, C=C, and C–N, respectively. The peaks at 1211 cm^−1^ and 1100 cm^−1^ are due to asymmetric and symmetric C–O–C stretching vibrations. The peaks between 2850 cm^−1^ and 2960 cm^−1^ arise from the C–H stretching vibrations of methyl/methylene. The wide absorption bands from 3150 cm^−1^ to 3400 cm^−1^ indicate the presence of amino (–NH_2_) and hydroxyl (–OH) functional groups.

The zeta potentials of GO, MOG, and CD particles were measured. It was found that particles of GO, MOG, and CDs had a negative charge with zeta potential values of −38.4 ± 1.12 mV; −33.2 ± 0.10 mV; and −41.7 ± 1.99 mV, respectively. The zeta potential values, as well as the elemental analysis data, indicate that MOG has the least stability in an aqueous suspension owing to its low content of functional groups. The CD solution exhibited the highest stability.

**Figure 3:**
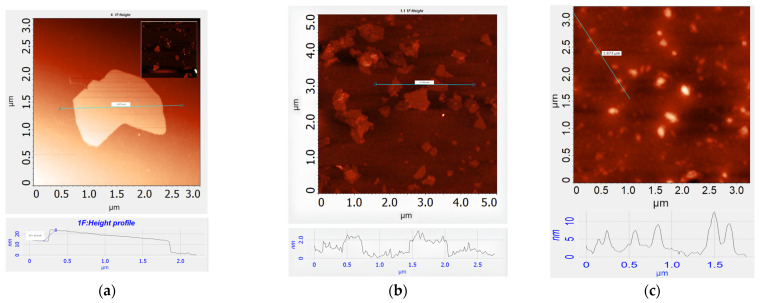
AFM images of (**a**) MOG; (**b**) GO; and (**c**) CDs.

Atomic force microscopy (AFM) (Figure 3) showed that the MOG particles are multigraphene flakes with a thickness of ~10 nm (~30 graphene layers). For comparison, the thickness of the GO flakes is approximately 1 nm. The average lateral dimensions of the flakes for the MOG ~2 µm and GO ~0.8 µm. AFM studies also showed that CDs consisted of particles with an average lateral size of ~200 nm and a thickness of 8.4 nm.

**Figure 4:**
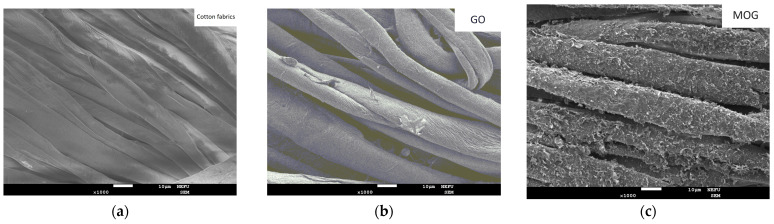

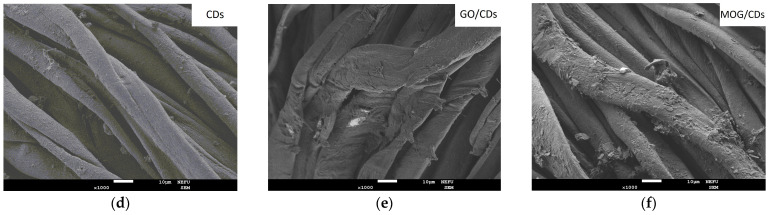
SEM images of cotton fibers at ×1000 magnification with deposited (**a**) pristine cotton fabrics; (**b**) GO; (**c**) MOG; (**d**) CD; (**e**) GO/CDs; and (**f**) MOG/CDs.

Scanning electron microscopy (SEM) images of the cotton fabrics decorated with carbon nanomaterials are shown in Figure 4. It can be seen that GO forms a uniform film on the surface of cotton fibers (Figure 4a). MOG and CDs (Figure 4b,c) tended to form agglomerates on the surface of the fibers. The mixture of the GO/CDs and MOG/CDs suspensions (Figure 4d,e) did not differ significantly from the GO and MOG films.

### 2.2. Comparison of the Antimicrobial Properties of GO, MOG, CDs, and Their Combinations

**Figure 5:**
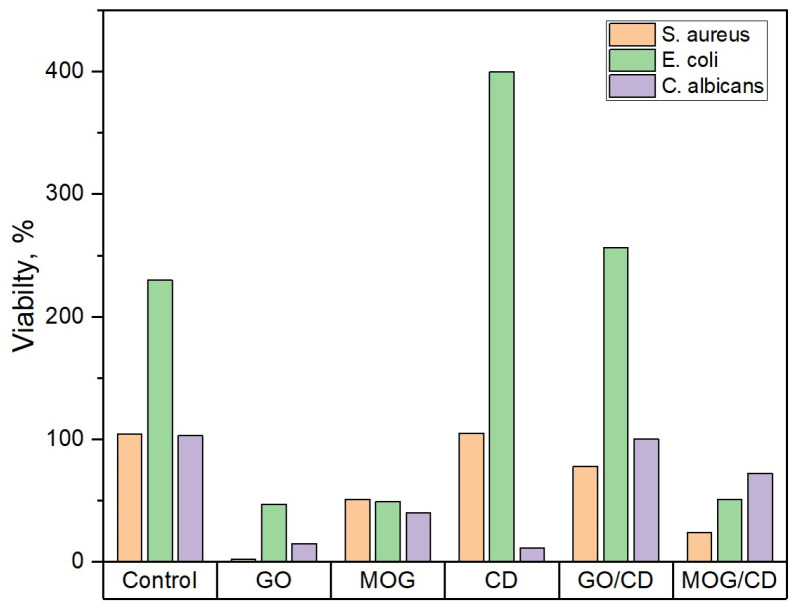
Survival rate (%) of microorganisms after 24 h of incubation with carbon nanomaterials.

According to the results of microbiological tests, suspensions of carbon nanomaterials exhibited different antimicrobial activities (Figure 5). GO showed a more pronounced antimicrobial activity than MOG, with the greatest effect against gram-positive bacteria. Thus, after 24 h of incubation of the test cultures with GO, the survival rate of microorganisms was as follows: *S. aureus*—2%, *E. coli*—47%, and *C. albicans*—15%. The survival rate of microorganisms during incubation with MOG was greater than that with GO: *S. aureus*—51%, *E. coli*—49%, and *C. albicans*—40%. The suspension containing CDs did not exhibit a significant antibacterial effect. However, antifungal effects were observed. The survival rates of test cultures incubated with CDs was *S. aureus*—105%, *E. coli*—400% and *C. albicans*—12%. The mixing of CDs with GO and MOG weakened their antimicrobial properties. The survival rates of the test cultures incubated with GO/CDs were significantly higher than those of the cultures incubated with GO: *S. aureus*—78%, *E. coli*—250%, and *C. albicans*—100%. Milder degradation of the antibacterial properties was observed for the MOG/CDs composite: *E. coli*—51% and *C. albicans*—72%. However, the addition of CDs to MOG significantly improved its antimicrobial effect against *S. aureus*—24%.

**Figure 6:**
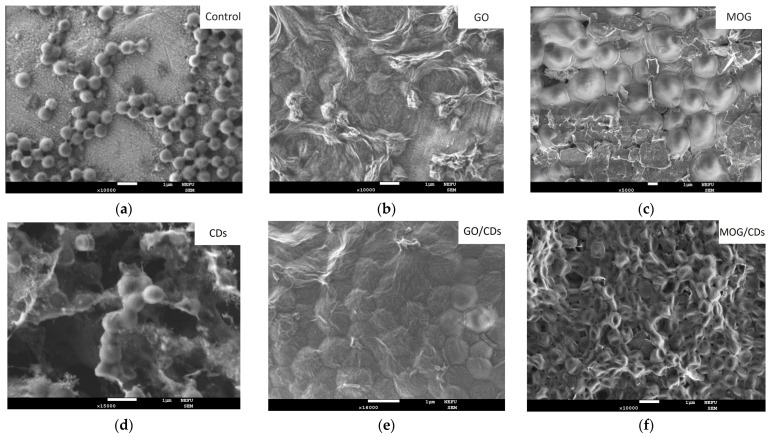
SEM images of test cultures of *S. aureus* dried on the SiO_2_ surface after 24 h of incubation with (**a**) the control; (**b**) GO; (**c**) MOG; (**d**) CD; (**e**) GO/CDs; and(**f**) MOG/CDs.

**Figure 7:**
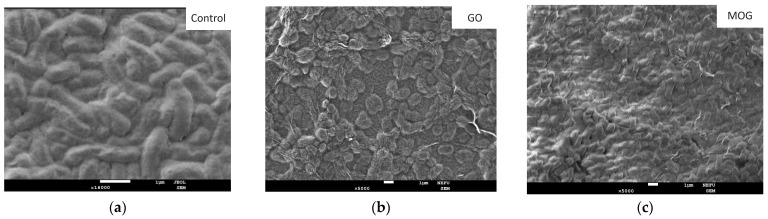

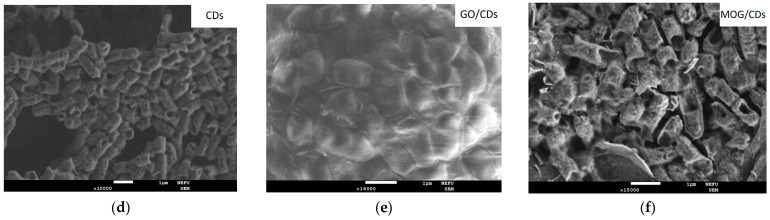
SEM images of test cultures of *E. coli* dried on the SiO_2_ surface after 24 h of incubation with (**a**) the control; (**b**) GO; (**c**) MOG; (**d**) CD; (**e**) GO/CDs; and (**f**) MOG/CDs.

**Figure 8:**
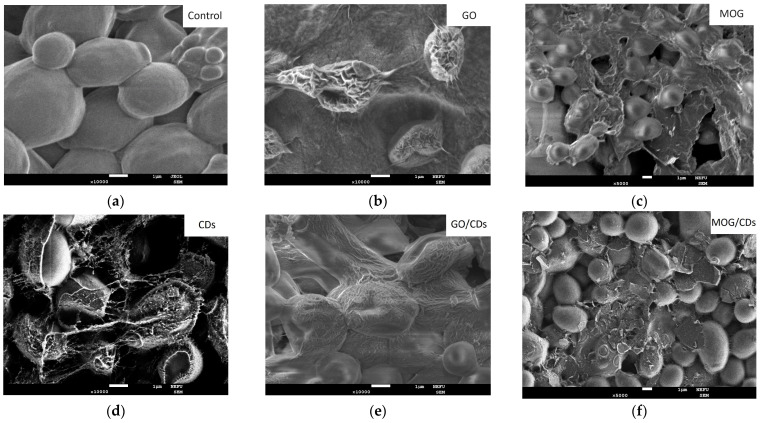
SEM images of test cultures of *C. albicans* dried on the SiO_2_ surface after 24 h of incubation with (**a**) the control; (**b**) GO; (**c**) MOG; (**d**) CD; (**e**) GO/CDs; and (**f**) MOG/CDs.

SEM images of cultures after intubation for 24 h in nanomaterial suspensions showed different effects on microbial cells. (Figure 6, Figure 7 and Figure 8). The images show a significant change in the morphology of microbial cells after incubation with GO. The surface of the cells had a wrinkled structure owing to the formation of the GO film. The cell wall of the bacteria was damaged and its shape was disrupted. However, no such effect was observed when incubated with MOG.

The cell membranes of the microorganisms appeared intact. However, bacterial cells were covered with MOG agglomerates. SEM images of the interaction of CDs with microorganisms showed an unchanged *S. aureus* morphology. It can be seen that the cell walls of *E. coli* have a deformed appearance. The cells of *C. albicans* are covered with a dense film, under which destructive processes probably occur. The appearance of such films upon interaction with CDs has not been observed in other cultures. The interaction of microorganisms with GO/CDs had a similar appearance but there were fewer altered cells and bacteria and fungi with a normal morphology were observed. After incubation with MOG/CDs, *S. aureus* lost its spherical shape, indentations and invaginations were visible in the SEM images, and no cells with normal morphology were present. The structure of *E. coli* was also disrupted when incubated with MOG/CDs; the cells appeared torn and had an irregular shape. The morphology of *C. albicans* was preserved. The cells are in a state of active growth and reproduction, whereas the bacterial cells are significantly damaged.

The data obtained when studying the antimicrobial effects of cotton textiles with deposited nanomaterials are presented in Table 2. These results generally correlate with the activity of the carbon nanomaterials suspensions. Textiles treated with MOG and GO exhibited good antimicrobial activity, which decreased when they were combined with CDs. The samples containing CDs did not exhibit significant antimicrobial activity. The combination of MOG/CDs improved its effect on *S. aureus* and *C. albicans*, while separate materials did not show significant antimicrobial activity toward it. After 10 cycles of washing, the antimicrobial properties of the textiles decreased in all cases (Table 3). The most significant reduction was observed for the CDs.

## 3. Discussion

The survival rate of microorganisms obtained from bacteriological examinations generally correlates with the changes visualized by SEM. Nanomaterials that did not show a significant antibacterial effect in survival tests on SEM showed preservation of cell morphology and the ability of the cultures to grow and reproduce. However, it is worth noting that to obtain images, suspensions of nanomaterials were dried on a SiO_2_ surface, which leads to interactions that are absent in a liquid medium, such as the creation of homogeneous thick films of nanomaterials. A comparison of the results of the current study with those in the literature is shown in Table 4. Overall, it is difficult to compare the results of cell vitality because of the high significance of concentration, chemical composition, lateral sizes, and experimental methodology on the antimicrobial activity of nanomaterials. In general, the obtained results correlate with the literature data. However, the high selectivity of the CDs used in this study toward C. albicans can be noted. This discrepancy with the literature data can be attributed to the differences in the concentrations and chemical compositions used.

The difference in the antimicrobial activity of materials in the form of suspensions can be explained by a combination of the effects of various factors. SEM studies suggest that the leading antimicrobial mechanism of GO is cell wrapping, which leads to the degradation of the microbial cell membrane and its deformation. This effect was also observed for the MOG. However, the wrapping was incomplete. Since gram-positive bacteria are more susceptible to cell disruption and have higher adhesion to GO [12], they are more sensitive to GO than gram-negative bacteria. The weaker effect of MOG suspensions may be due to the greater thickness of the flakes compared to GO (~10 nm and ~1 nm, respectively). In addition, the higher tendency of MOG to agglomerate in an aqueous suspension due to the low oxygen content and surface charge (−33.2 ± 0.10 against 38.4 ± 1.12 for GO) leads to incomplete wrapping of the cells. It is known that agglomeration of graphene particles leads to deterioration of the antibacterial effect [11]. In addition, MOG has a lower oxygen content than GO, which can significantly decrease the effect of oxidative stress on microbial cells. The roles of functional groups in GO and MOG are unclear. The main difference in the functional group content is the presence of carboxyl groups in the GO. A higher concentration of carboxyl groups leads to better adhesion to the bacterial cell surface [46]. This may be one of the factors that lead to better cell wrapping of GO compared to MOG, which was corroborated by SEM. However, it should be noted that other factors may also play a role in antimicrobial activity, such as better dispersibility, which was better assessed by zeta potential studies. The combination of these factors can explain the poor antimicrobial effect of MOG compared to that of GO. In contrast, CDs have a high zeta potential and dispersibility. As previously mentioned, functional groups play a major role in the antimicrobial activity of CDs [23]. It has been shown [24] that functionalization of CDs with nitrogen groups increases the antimicrobial effect against gram-positive B. subtilis. However, for gram-negative E. coli, there was no significant change in the inhibition. This may be one of the reasons why the CDs were not selective against E. coli in this study. The weak antimicrobial effect of CDs can also be attributed to the fact that a high negative charge on the surface helps to reduce the adhesion of bacteria due to the negative charge on their membrane [2]. This effect was indirectly confirmed by the fact that the CDs exhibited a more pronounced antifungal effect against *C. albicans*. The membrane of *C. albicans* has a much lower negative charge than that of the bacteria [47]. Similarly, adding CDs to suspensions with GO and MOG reduces their activity because of the addition of an additional negative charge that repels bacterial cells. SEM studies also revealed the formation of a film on the surface of *C. albicans* when incubated with the CDs. This effect was not observed in bacterial cells and requires further research.

In the case of decorated cotton textiles, it can be seen that GO has higher antimicrobial activity and washing stability than other tested materials. This may be due to the more uniform and stable film of GO on the surface of the fibers, which can be observed in the SEM images. The MOG exhibited a less pronounced antimicrobial effect. Cell wrapping is unlikely to occur in the dry surface media of textiles with deposited nanomaterials. A more pronounced effect was caused by oxidative stress and cell surface damage on sharp edges. GO has a higher oxygen content than MOG, which can contribute to oxidative stress in microbial cells on the surface of the fibers. In addition, MOG tends to form macroscopic agglomerates, which significantly reduce cell surface damage. The weak effect of fabrics with CDs is most likely due to their agglomeration into large macroscopic particles upon drying, during which they lose their properties [48]. The deterioration of the properties of GO/CDs can be explained by the CD particles preventing the interaction between the bacterial cells and the smooth GO surface. In contrast, the antimicrobial properties of MOG/CD textiles were superior to those of the original materials. This can be attributed to the MOG preventing the agglomeration of CDs upon drying, thus providing a matrix for CDs. CDs in the MOG matrix exhibit more oxidative stress to bacterial cells than GO/CDs because of their more developed surface morphology, which can be seen in the SEM images. The differences in the effects of textiles with GO and MOG on gram-positive and gram-negative bacteria and the synergetic effect of MOG/CDs deposited on textiles require further research using more accurate methods. Washing stability tests showed a significant reduction in the antimicrobial properties of all tested materials owing to the degradation of the deposited films. CDs show less washing stability owing to their high dispersibility and poor adhesion to the surface of cotton fibers.

## 4. Materials and Methods

### 4.1. GO and MOG Synthesis

An aqueous suspension of GO was prepared using a modified Hummer’s method described in our previous study [49]. In brief, 0.1 g of flake graphite powder (Sigma Aldrich, St. Louis, MO, USA) was placed in 14 mL of concentrated sulfuric acid (Rushim, Moscow, Russia) with the 0.4 g of potassium permanganate (Rushim, Moscow, Russia). The resulting reaction mixture was placed in a beaker and stirred for three weeks. The reaction was stopped with a 5% solution of hydrogen peroxide (Rushim, Moscow, Russia) (7 mL) and washed with deionized water until the filtrate became neutral. A brown gel-like mass was diluted with water (50 mL) and treated with ultrasound for 5 min on a disperser Hielscher Up 200 St (Hielscher Ultrasonics, Teltow, Germany) at 60 W.

An aqueous suspension of MOG was obtained by the electrochemical exfoliation of graphite. A detailed description of the process was provided in our previous study [50]. Briefly, a graphite rod (Polyprof-L LLC, Moscow, Russia) was electrochemically exfoliated in an NH_4_SO_3_ (Rushim, Moscow, Russia) solution at 10 V. The resulting product was washed with distilled water and dispersed in water by using an ultrasonic disperser Hielscher Up 200 St (Hielscher Ultrasonics, Teltow, Germany) at 30 W for 10 min.

### 4.2. Synthesis of CDs

The CDs were synthesized from citric acid using a solvothermal method. In total, 4.3 g of citric acid (Chip and Dip, Moscow, Russia) and 8.6 g of urea (Vostokreaktiv, Khabarovsk, Russia) were dissolved in 65 mL of formamide (Reakhim, Moscow, Russia) using ultrasonic disperser Elmasonic S40H Elma (Elma Schmidbauer GmbH, Singen, Germany). The clear solution was then transferred to a 90 mL stainless steel polytetrafluoroethylene autoclave (TOPTION Laboratory Store, Xi’an, China) and heated for 12 h at 180 °C. Upon completion of the synthesis, the autoclave was cooled overnight to room temperature. The solution was transferred to a dialysis bag with pores (MWCO) of 12–14 kDa (Spectrum Labs, Moscow, Russia) and placed in a beaker containing deionized water and a magnetic stirrer HS Pro-DT (Stegler, Moscow, Russia) for 24 h. The solution was then filtered using a track membrane with a pore size of 100 nm (JINR, Dubna, Russia). The resulting product was centrifuged using an Eppendorf MiniSpin plus (Eppendorf AG, Hamburg, Germany) at 14,500 rpm for 5 min and decanted over the sediment.

### 4.3. Preparation of Suspensions and Textiles Decorated with GO, MOG, and CDs

**Figure 9:**
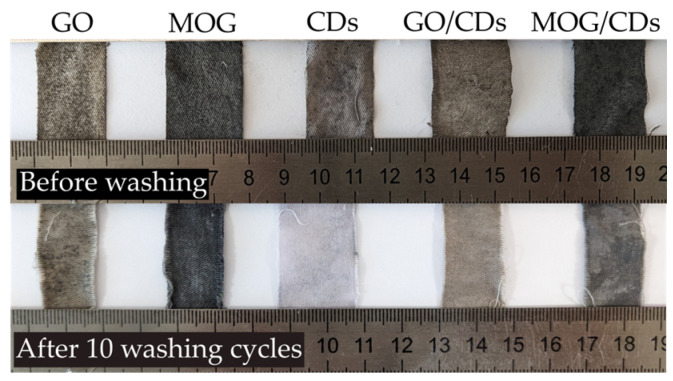
Cotton textile samples coated with carbon nanomaterials before and after 10 washing cycles.

The suspensions were brought to a concentration of 1 mg/mL by diluting previously obtained suspensions. The concentration was determined by weighing the dry residue using Vibra AF-R220CE (Shinko Denshi Co. ltd., Tokyo, Japan). Suspensions of GO/CDs and MOG/CDs were obtained by mixing the suspensions at a ratio of 1:1, followed by ultrasonic treatment at a power of 30 W for 30 min using Hielscher Up 200 St (Hielscher Ultrasonics, Teltow, Germany). Cotton fabrics (bleached calico with a density of 120 g/m^2^, Ivanovo Garment Factory, Ivanovo, Russia) were used as the base material. Textile samples were dipped into aqueous suspensions of GO, MOG, CDs, GO/CDs, and MOG/CDs at a concentration of 1 mg/mL and treated with mild ultrasound for 30 min at room temperature. The fabrics were then dried under normal conditions until they were completely dry. To evaluate the washing stability, the samples were washed according to GOST 9733.4-83 [51]. The samples were placed in a washing solution that consisted of 0.5 g of soap (Belgorod soap facroty, Belgorod, Russia), 0.2 g of soda (Bashkirskaya Sodovaya Kompaniya, Sterlitamak, Russia), and 100 mL of water. The detergent solution in the beaker containing the samples was stirred at 300 rpm using an magnetic stirrer HS Pro-DT (Stegler, Moscow, Russia) at 60 °C for 30 min. Photographs of the fabrics before and after washing are shown in Figure 9.

### 4.4. Research Methods

The synthesized carbon nanomaterials were studied by Raman spectroscopy at the Integra Spectra installation (NT–MDT, Zelenograd, Russia) using a green laser with a wavelength λ = 532 nm, an Andor grating spectrum of 600 l/mm, and a resolution of 1 cm^−1^. The study of the surface morphology and determination of the thickness of individual GO and MOG flakes were carried out using an AFM Solver Next (NT–MDT, Zelenograd, Russia) in non-contact scanning mode with high-resolution silicon AFM Cantilevers NSG10 (TipsNano Co., Tallinn, Estonia) with 240 kHz resonant frequency and a constant 11.8 N/m force. To study the composition of the functional groups of the GO, MOG, and CDs films, IR spectra were measured using a Spotlight 200i setup (PerkinElmer, Waltham, MA, USA) in the range of 550–4000 cm^−1^, with a resolution of 8 cm^−1^. The zeta potentials of GO, MOG, and CD particles were measured using a Zetasizer Nano ZS (Malvern Panalytical, Malvern, UK). A JEOL-7800F (Jeol, Tokyo, Japan) SEM unit operated at 3 kV was used to study the morphology of the nanomaterials deposited on the fabrics surfaces and the microbial reactions to the nanoparticles. Elemental analysis of the films was performed using energy-dispersive spectroscopy with a NanoAnalysis microanalysis system (Oxford Instruments, Oxford, UK) attached to the SEM. EDS was performed at ×1000 magnification, 5 kV, and 1.2 nm spatial resolution.

### 4.5. Methods for Testing the Antimicrobial Activity of Suspensions of Carbon Nanomaterials and Decorated Textiles

Reference strains from the American Collection of Type Cultures were used as test cultures: *Escherichia coli* ATCC^®^ 10536, *Staphylococcus aureus* ATCC^®^ 6538, and *Candida albicans* ATCC^®^ 10231.

To test the antimicrobial properties, 50 μL suspensions of the following carbon nanomaterials were added to the wells of the immunological tablet: GO, MOG, CDs, MOG/CDs, GO/CDs, and distilled water as a control. Next, 10 μL of the test culture suspension was added to each well. Sowing on nutrient media was performed immediately after mixing and after 24 h of incubation with carbon nanomaterials. The test cultures were cultivated on a meat-peptone agar medium. Sabouraud agar was used for fungi of the genus *Candida*.

To prepare samples for SEM, a drop (0.1 mL) of a suspension of carbon nanomaterials intubated with microbial culture was applied to a thoroughly washed SiO_2_ substrate (Telecom-STV, Moscow, Russia).

To determine the antimicrobial activity of treated textiles, the absorption method from the national standard of the Russian Federation “Determination of the antibacterial activity of products with antibacterial treatment: textile materials” GOST R ISO 20743-2012 was used [52]. Textile samples were placed in sterile Petri dishes. An aqueous suspension of 200 µL of the test culture at a concentration of 105 CFU/mL was carefully applied to the textile to avoid contact with the container surface. Then, the textile sample was shaken, transferred into a sterile Eppendorf tube, and filled with 1000 μL of liquid nutrient medium. The solid nutrient medium was sowed immediately after mixing and after 24 h of incubation at 37 °C. Seeding was performed according to the Tsarev-Melnichenko method followed by colony counting.

The antimicrobial activity of the textiles with deposited nanoparticles was calculated according to the method specified in the standard. The growth level in the control sample (F) was determined using the following formula:

F = lgC_t_ − lgC_0_, (1)

where lgC_t_ is the decimal logarithm of the number of bacteria obtained from the test sample of the control fabric after incubation for 18–24 h and lgC_0_ is the decimal logarithm of the bacterial count obtained from the control fabric test sample immediately after transfer to the control fabric. The antimicrobial activity of the test sample (A) was obtained using the following formula:

A = (lgC_t_ − lgC_0_) − (lgT_t_ − lgT_0_) = F − G, (2)

where A is the value of antimicrobial activity; F is the level of growth on the control sample; G is the level of growth on the sample that has undergone antimicrobial treatment; lgT_t_ is the value of the decimal logarithm of the number of bacteria obtained from the test sample after incubation for 18–24 h; and lgT_0_ is the value of the decimal logarithm of the number of bacteria obtained from the test sample immediately after transfer.

## 5. Conclusions

The data obtained indicate the pronounced antimicrobial activity of GO and MOG associated with their ability to damage the cell wall of bacteria and the membrane of fungi by wrapping. CDs, in the form of suspensions, showed better effectiveness against *C. albicans* but had no effect on gram-positive or gram-negative bacteria, which can be attributed to the high surface charge of CDs, which repels bacterial cells. The combination of CDs with GO and MOG significantly weakened the antimicrobial activity of the latter. However, the combination of MOG and CDs showed a synergetic effect on antimicrobial activity toward gram-positive *S. aureus*. Studies on antimicrobial activity have demonstrated the effectiveness of GO and MOG deposited on cotton textiles. MOG-treated samples showed no activity toward *S. aureus*. Textiles showed a greater effect against the thin-walled gram-negative bacterium *E. coli*. The synergetic antimicrobial effect of the MOG and CDs combination on the textile surface was revealed. Research has shown that the structural and physicochemical properties of original nanomaterials play a significant role. Differences in chemical composition and morphology can lead to significant changes in the antimicrobial effects.

## Figures and Tables

**Table 1:** Atomic content of elements according to EDS in GO, MOG, and CDs.

Element	GO	MOG	CDs
C, %	58.6	93.9	37.7
O, %	41.4	6.1	33.5
N, %	-	-	28.8

**Table 2:** Antimicrobial activity of treated cotton textile A.

Microorganism	GO	MOG	CDs	GO/CDs	MOG/CDs
*S. aureus*	2	0	0	1	1
*E. coli*	2	2	1	1	1
*C. albicans*	1	1	0	1	2

**Table 3:** Antimicrobial activity of treated cotton textile A after 10 washing cycles.

Microorganism	GO	MOG	CDs	GO/CDs	MOG/CDs
*S. aureus*	1	−1	0	0	1
*E. coli*	1	1	−4	0	0
*C. albicans*	1	1	0	1	−1

**Table 4:** Comparison of antibacterial properties of GO, MOG, and CDs.

Microbial Culture		Cell Vitality, %	
	GO	MOG	CDs
*S. aureus*	2 *, 60 [11], 8 [12], 6.3 [14]	51 *, 0 [33]	100 *, 0 [41]
*E. coli*	47 *, 8 [15], 90 [11], 33.3 [12]	49 *, ~100 [33]	100 *, 1 [23], ~100 [24], 0 [41]
*C. albicans*	15 *, ~20 [42], 28 [43]	40 *	12 *, 90 [44], 60 [45]

* Current study.

## Data Availability

The data presented in this study are available on request from the corresponding author. The data are not publicly available due to the data protection policy of the university.
